# Serum lipidomic profiling as a useful tool for screening potential biomarkers of hepatitis B-related hepatocellular carcinoma by ultraperformance liquid chromatography–mass spectrometry

**DOI:** 10.1186/s12885-015-1995-1

**Published:** 2015-12-18

**Authors:** Ana Maria Passos-Castilho, Valdemir Melechco Carvalho, Karina Helena Morais Cardozo, Luciana Kikuchi, Aline Lopes Chagas, Michele Soares Gomes-Gouvêa, Fernanda Malta, Ana Catharina de Seixas-Santos Nastri, João Renato Rebello Pinho, Flair José Carrilho, Celso Francisco Hernandes Granato

**Affiliations:** Division of Infectious Diseases, Federal University of Sao Paulo, 781 Pedro de Toledo Street, 15th floor, Sao Paulo, SP 04039032 Brazil; Fleury Group, Sao Paulo, SP Brazil; Department of Gastroenterology, Sao Paulo Clinicas Liver Cancer Group, Instituto do Câncer do Estado de São Paulo, Hospital das Clínicas, Sao Paulo, SP Brazil; Department of Infectious Diseases, University of São Paulo School of Medicine, Sao Paulo, SP Brazil

**Keywords:** Biomarker, Hepatocellular carcinoma, Hepatitis B, Lipidomics, UPLC-MS, Diagnosis

## Abstract

**Background:**

Chronic hepatitis B (CHB) virus infection is a major cause of hepatocellular carcinoma (HCC), as late diagnosis is the main factor for the poor survival of patients. There is an urgent need for accurate biomarkers for early diagnosis of HCC. The aim of the study was to explore the serum lipidome profiles of hepatitis B-related HCC to identify potential diagnostic biomarkers.

**Methods:**

An ultraperformance liquid chromatography mass spectrometry (UPLC-MS) lipidomic method was used to characterize serum profiles from HCC (*n* = 32), liver cirrhosis (LC) (*n* = 30), CHB (*n* = 25), and healthy subjects (*n* = 34). Patients were diagnosed by clinical laboratory and imaging evidence and all presented with CHB while healthy controls had normal liver function and no infectious diseases.

**Results:**

The UPLC-MS-based serum lipidomic profile provided more accurate diagnosis for LC patients than conventional alpha-fetoprotein (AFP) detection. HCC patients were discriminated from LC with 78 % sensitivity and 64 % specificity. In comparison, AFP showed sensitivity and specificity of 38 % and 93 %, respectively. HCC was differentiated from CHB with 100 % sensitivity and specificity using the UPLC-MS approach. Identified lipids comprised glycerophosphocolines, glycerophosphoserines and glycerophosphoinositols.

**Conclusions:**

UPLC-MS lipid profiling proved to be an efficient and convenient tool for diagnosis and screening of HCC in a high-risk population.

**Electronic supplementary material:**

The online version of this article (doi:10.1186/s12885-015-1995-1) contains supplementary material, which is available to authorized users.

## Background

Hepatitis B virus (HBV) infection is one of the main causes of chronic liver disease worldwide. It is estimated that 240 million individuals are chronically infected with HBV [1]. Depending on the presence of co-factors, progression to liver cirrhosis (LC) may occur at a rate of 2 to 10 % per year, whereas hepatocellular carcinoma (HCC) may develop in 2–4 % of patients per year. HBV is estimated to be responsible for 30 % of cirrhosis- and 45 % of HCC-related deaths [2].

In Brazil, HBV accounts for 13–25 % of HCC cases in most geographical regions, reaching 40 % of HCC cases in the Mid-west [3].

HCC is a complex and heterogeneous tumor with several genomic alterations and its incidence has been increasing worldwide. It is the sixth most common cancer and the second cause of cancer-related death. When diagnosed at an early stage, surgical options such as resection or liver transplantation, or local ablative therapies, can be applied with intent to cure HCC. However, until now, no effective serum or plasma biomarkers have been found for accurate screening or diagnosis of HCC [4, 5].

HCC diagnosis is most commonly performed by ultrasound examination, CT scan and/or magnetic resonance. Histopathology confirmation may also be necessary in some cases. However, there are some limitations related to risk of complications and feasibility of the biopsy due to tumor location. Moreover, the effectiveness of ultrasound for early detection of HCC is highly dependent on the stage of liver fibrosis, the quality of the equipment and the expertise of the operator [4].

Alpha-fetoprotein (AFP) is the most widely used biomarker for HCC. However, its sensitivity is only up to 60 % and elevated AFP levels are also common in LC and chronic liver disease [6]. Thus, there is an urgent need to identify better HCC biomarkers.

The ideal biomarker should be specific and able to discriminate HCC from regenerative nodules irrespective of the stage of liver disease. Furthermore, the biomarker should be sensitive, allowing detection at an early stage, and should be easily measurable, reproducible, and minimally invasive.

Recently developed mass spectrometry (MS)-based techniques such as lipidomics are promising tools for the discovery and subsequent identification of molecules associated with various diseases. Separation techniques, like ultraperformance liquid chromatography (UPLC), coupled to MS enable the analysis of complex samples such as plasma or serum with very high sensitivity and accuracy [7, 8].

Once lipid biomarkers are identified through UPLC-MS, they can be later investigated in clinical laboratory routine using simple and accessible colorimetric and/or enzymatic techniques. Nonetheless, studies on lipid profiling and fingerprinting of HCC are still scarce [9–12].

The aim of this study was to assess the serum lipid patterns of HCC by performing UPLC-MS to search for potential biomarkers for diagnosis in HBV chronic infected patients (HBV-HCC).

## Methods

### Study design, sample and data collection

A total of 87 patients with chronic hepatitis B (CHB) were enrolled from 2012 to 2014 at the Hospital das Clínicas of the University of Sao Paulo School of Medicine, including 32 patients with HBV-HCC, 30 patients with HBV-LC and 25 patients with CHB. Additionally, 34 eligible blood donors with normal liver function and no infectious diseases were recruited at COLSAN Beneficent Association for Blood Collection to serve as healthy controls. CHB was diagnosed based on the presence of HBsAg for at least 6 months. LC was diagnosed by histopathology, clinical features and/or elastography and HCC was diagnosed using imaging or histopathology techniques, in accordance with guidelines of the Brazilian Society of Hepatology.

Blood samples were obtained by venipuncture and drained into blood collection tubes. The samples were centrifuged immediately after collection and serum was stored at −80 °C until analysis.

Demographic, clinical and laboratory data were collected from medical records. The study was approved by the ethics committee of human research of the Federal University of Sao Paulo and the University of São Paulo School of Medicine (2012/81656 and 2014/569922) and all patients gave written informed consent.

### Extraction of lipids

Lipids were extracted from each sample using a modified Bligh-Dyer protocol [13]. Immediately after thawing, 100 μL of serum were dissolved in 850 μL of a mixture of water/chloroform/methanol (1:2.5:5, v/v) and vortexed well for 5 min. After vortexing, 250 μL of chloroform were added and the tubes were agitated for 15 min at 700 rpm. Then, 200 μL of deionized water were added and the tubes were centrifuged at 14,000 rpm for 15 min at room temperature. Following this protocol a 2-phase system (aqueous top, organic bottom) was achieved. The bottom phase containing lipids was gently recovered using a micropipette, dried, and resuspended in 350 μL of acetonitrile/water (3:2, v/v). All chemicals were of analytical reagent grade and used as received.

### UPLC-MS analysis

Reversed-phased analysis was performed on a Waters ACQUITY IClass UPLC system equipped with a Waters Acquity CSH C18 1.7 μm x 2.1 × 100 mm column coupled to a Waters Synapt-MS hidrid quadrupole-time of flight mass spectrometer operating in the positive ion electrospray mode. A mass scan range of 200 to 1,200 mass-to-charge ratio (*m/z*) was set for data acquisition in continuous mode with optimized parameters for ionization and mass transmission. Acetonitrile/water (3:2, v/v) was used as mobile phase A and isopropanol/acetonitrile (9:1, v/v) was used as mobile phase B, both with 10 mM ammonium formate and 0.1 % formic acid as additives. The flow rate was set at 600 μL/min and the injection volume was 10 μL. A binary gradient was optimized as follows: the composition of mobile phase B was changed from 15 % to 30 % in 2 min, then to 48 % in 30 s and reached 82 % in 8.5 min. Subsequently, it was changed to 99 % in 30 s, held for another 30 s and then dropped to 15 % in 6 s prior to being held until a total run time of 15 min. The mass spectrometer was previously calibrated with 0.1 % phosphoric acid in water/acetonitrile (1:1, v/v) and a solution of 0.5 ng/μL leucine enkephalin in water/acetonitrile (1:1, v/v) with 0.1 % formic acid infused in the reference probe at a flow rate of 5 μL/min was used as lock mass spray at a 30 s frequency for accurate mass determination. All analyses were acquired using the lock spray and the instrument was recalibrated every 4 run-hours to ensure accuracy and reproducibility. Furthermore, a quality control of pool plasma samples was analyzed after every 10 runs, and 10 peaks well distributed from 200 to 1,200 *m/z* were assessed.

### Data pretreatment and statistical data analyses

All data obtained from the UPLC-MS analyses were processed with the Waters Progenesis software (Manchester, UK). This step included mass correction, chromatograms and spectra alignment and peak detection using default parameters. After attribution, the matrix of the features characterized by their *m/z* and retention time (RT) was uploaded into the MetaboAnalyst 3.0 (The Metabolomics Innovation Centre, Canada). For normalization, data was mean-centered and divided by the square root of standard deviation of each variable (Pareto scaling).

For multivariate analysis, the unsupervised principal component analysis (PCA) was first utilized in all samples (Additional file 1). Supervised partial least-squares-latent structure discriminate analysis (PLS-DA) was then performed to identify biomarkers that contributed to the clustering. Validation with a permutation test and 100 repetitions was performed to prevent model overfitting. Potential biomarkers that differentiated HCC from LC, CH and healthy subjects (HS) were selected based on the variable importance in the projection (VIP) values and univariate statistical significance after Mann–Whitney test and fold-change analyses. Receiver operating characteristic (ROC) curves were performed to evaluate the accuracy of the potential biomarkers and the proposed model using the ROCCET (The Metabolomics Innovation Centre, Canada).

Statistical analyses of demographic, clinical and laboratory data of subjects were performed using SPSS version 11.0 (SPSS Inc., Chicago, IL, USA). Descriptive statistics consisted of the characterization of the studied population (demographic, clinical and laboratory characteristics) through the respective percentages or mean/median and standard deviation (SD) for continuous variables. Bivariate analysis consisted of Fisher exact test to compare categorical values. For continuous variables, Student’s *t*-test was use to compare means of normally distributed variables, while non-normally distributed variables were subjected to Mann–Whitney *U* test. Statistical significance level was *P* < 0.050. All reported values are 2-tailed.

A tentative identification of the differentiating lipids was performed on the LIPID MAPS and HMDB databases.

## Results

The mean age of patients was 59.0 years old in the HCC group, 56.8 in the LC group, and 37.1 in the CHB group. The mean age of the HS was 42.6 years. In the HCC group 81.3 % of patients were males, while in the LC, CH and HS groups they were 66.7, 68.0 and 38.2 %, respectively (Table 1).

**Table 1:** Demographic data of the enrolled population of the study by group

Characteristics	HS	CH	LC	HCC	*P*
Number	34	25	30	32	-
Mean age ± SD	42.6 ± 14.8	37.1 ± 14.2	56.8 ± 11.0	59.0 ± 11.3	0.447
Median age	42.0	35.0	56.5	57.0	0.812
Age range	21–67	19–63	34–80	38–85	-
Gender (M/F)	13/21	17/8	20/10	26/6	0.190

*HS* healthy subjects, *CH* chronic hepatitis, *LC* liver cirrhosis, *HCC* hepatocellular carcinoma, *SD* standard deviation, *M* male, *F* female

Clinical and laboratory data analyses were performed for the HCC and LC groups (Table 2). In summary, the mean levels of AFP, alkaline phosphatase (ALP), and gamma-glutamyl transpeptidase (GGT) were significantly higher in the HCC group, while prothrombin time (PT) was lower. Nonetheless, the Child-Pugh score distribution was only slightly different between the LC and the HCC groups, which presented 4 and 2 patients with B and C scores, respectively. Twenty-eight of the 32 HCC patients (87.5 %) presented with LC. HCC was classified as BCLC—Barcelona Clínic Liver Cancer staging system very early or early stage in 19 of the 32 cases (59.4 %), intermediate stage in 10 (31.2 %) and advanced or terminal stage in only 3 (9.4 %) cases (Additional file 2.).

**Table 2:** Clinical and laboratory data of patients with liver cirrhosis and hepatocellular carcinoma

Characteristics	LC	HCC	*P*
AFP (ng/mL)	6.2 ± 15.8	507.8 ± 1565.9	< 0.001^*^
AFP ≥ 20 ng/mL	2 (6.67)	12 (37.5)	-
AFP ≥ 200 ng/mL	0 (0.0)	6 (18.8)	-
ALT (UI/mL)	35.4 ± 41.3	39.5 ± 43.2	0.698
AST (UI/mL)	37.3 ± 22.4	45.3 ± 43.0	0.746
ALP (U/L)	86.6 ± 51.6	119.2 ± 70.1	0.022^*^
GGT (U/L)	64.2 ± 98.2	108.1 ± 121.0	0.045^*^
Total bilirubin (mg/dL)	0.7 ± 0.3	1.1 ± 1.4	0.481
Albumin (g/dL)	4.7 ± 0.4	4.4 ± 0.8	0.117
Platelets (*1,000/mm^3^)	155.8 ± 89.5	156.0 ± 86.3	0.910
PT (seconds)	14.1 ± 2.1	13.2 ± 1.7	0.013^*^
Child-Pugh score			
A	30 (53.6)	26 (46.4)	0.030^*^
B or C	0 (0.0)	6 (100.0)	
Total cholesterol (mg/dL)	179.4 ± 38.1	172.8 ± 41.0	0.511
Tumor size (mm)	-	37.0 ± 23.0	-
BCLC stage			
0	-	5 (15.6)	-
A	-	14 (43.8)	-
B	-	10 (31.2)	-
C	-	1 (3.1)	-
D	-	2 (6.3)	-

Results are presented as number and percentage for categorical variables and as mean value and standard deviation for continuous variables. *LC* liver cirrhosis, *HCC* hepatocellular carcinoma, *AFP* alpha-fetoprotein, *ALT* alanine aminotransferase, *AST* aspartate aminotransferase, *ALP* alkaline phosphatase, *GGT* gamma glutamyl transpeptidase, *PT* prothrombin time, *BCLC* Barcelona-Clinic Liver Cancer. *Significant at 0.05

**Fig. 1:**
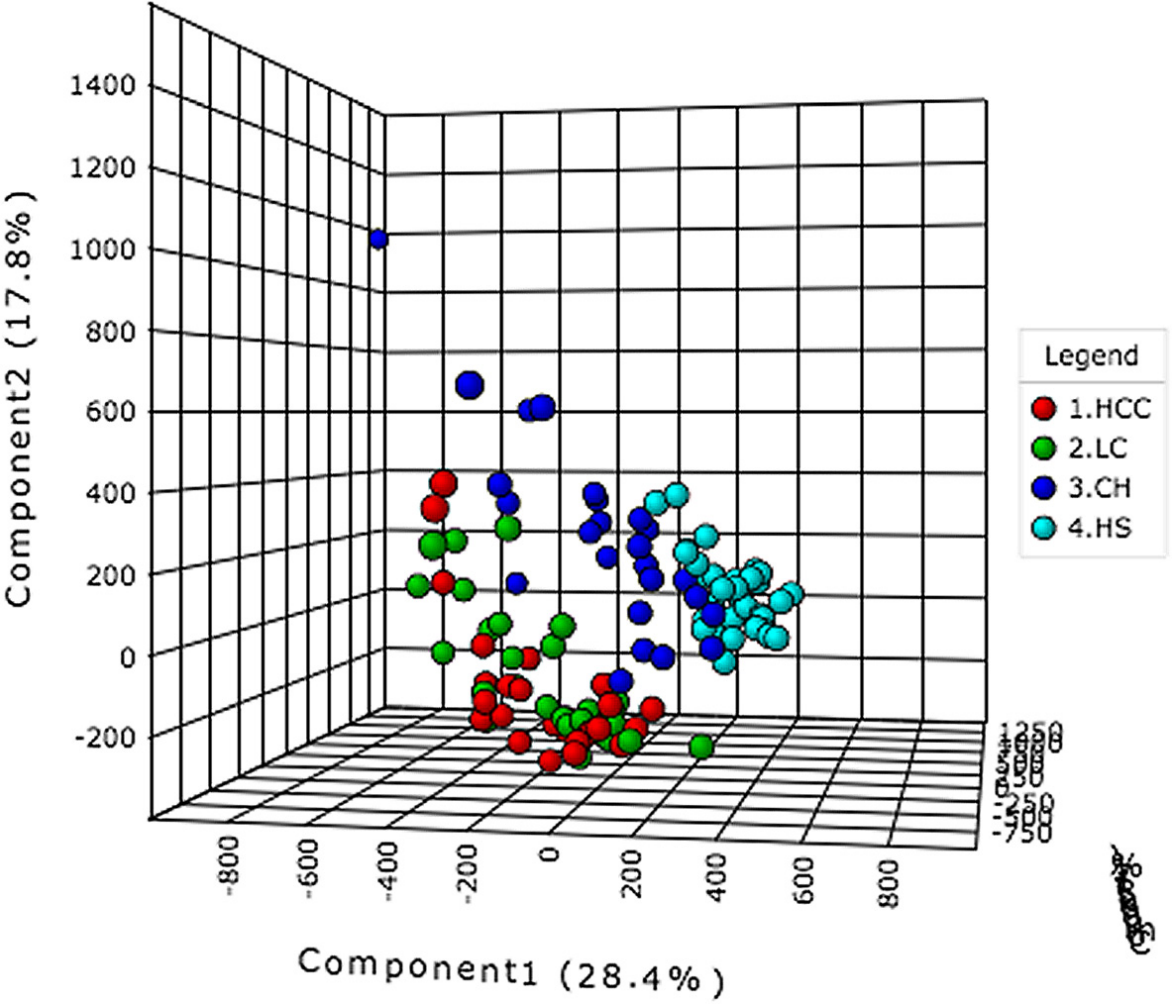
PLS-DA scores plot based on the UPLC-MS profiling data for the studied groups. Detailed legend: The score plots show the first, second and third latent variables. Each dot in the plot represents a patient according to its group. HCC, hepatocellular carcinoma; LC, liver cirrhosis; CH, chronic hepatitis; HS, healthy subjects

**Fig. 2:**
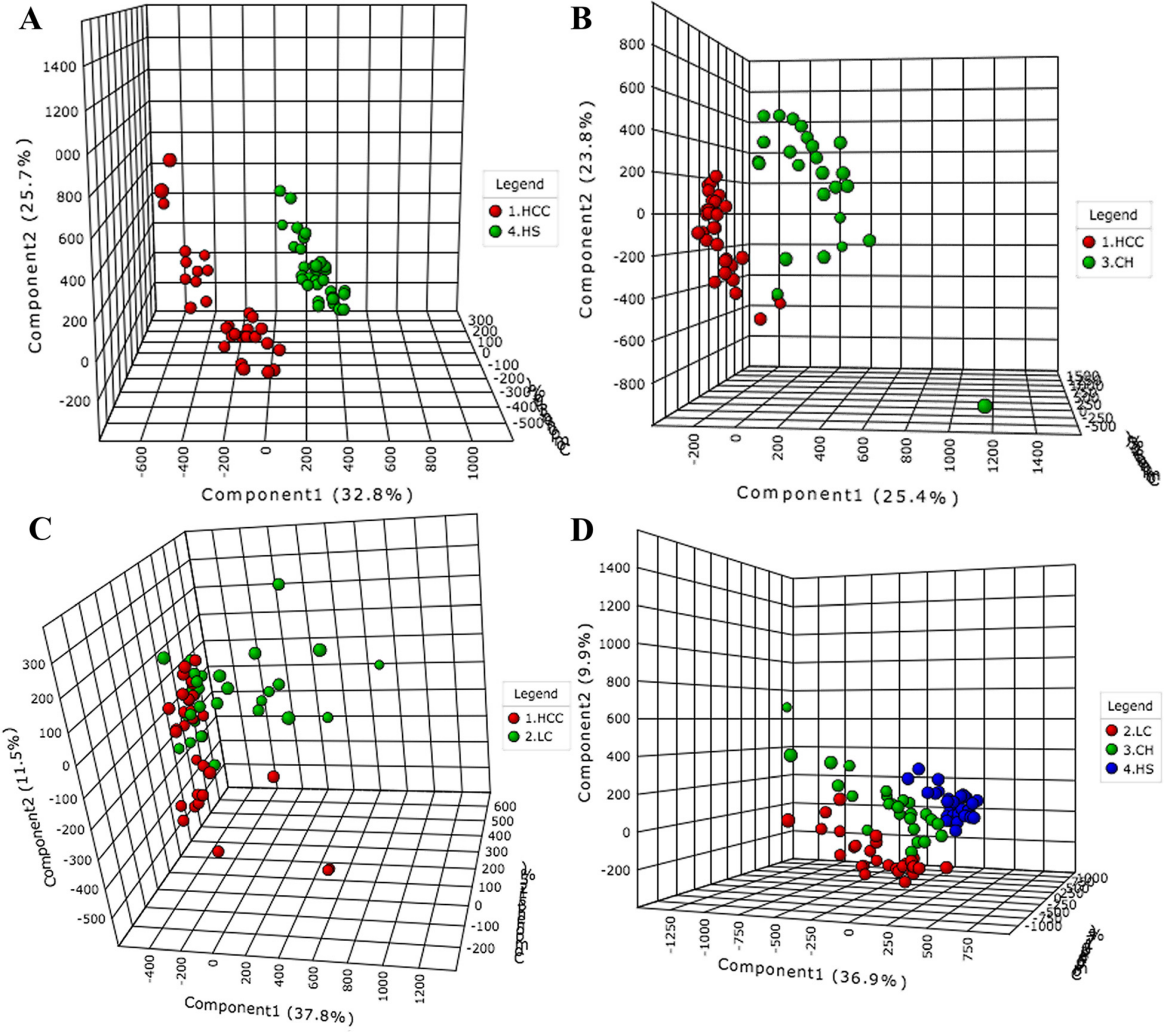
PLS-DA scores plot based on the UPLC-MS profiling data for (**a**) HCC versus HS; (**b**) HCC versus CH; (**c**) HCC versus LC; (**d**) LC versus CH versus HS. Detailed legend: The score plots show the first, second and third latent variables for each plot. Each dot in the plot represents a patient according to its group. HCC, hepatocellular carcinoma; LC, liver cirrhosis; CH, chronic hepatitis; HS, healthy subjects

A total of 2,698 ions were detected using the UPLC-MS method in this study. Figure 1 shows the PLS-DA score plot for the 4 groups evaluated. The separate PLS-DA score plots for inter-group comparisons are shown in Fig. 2.

### Hepatocellular carcinoma versus liver cirrhosis

**Fig. 3:**
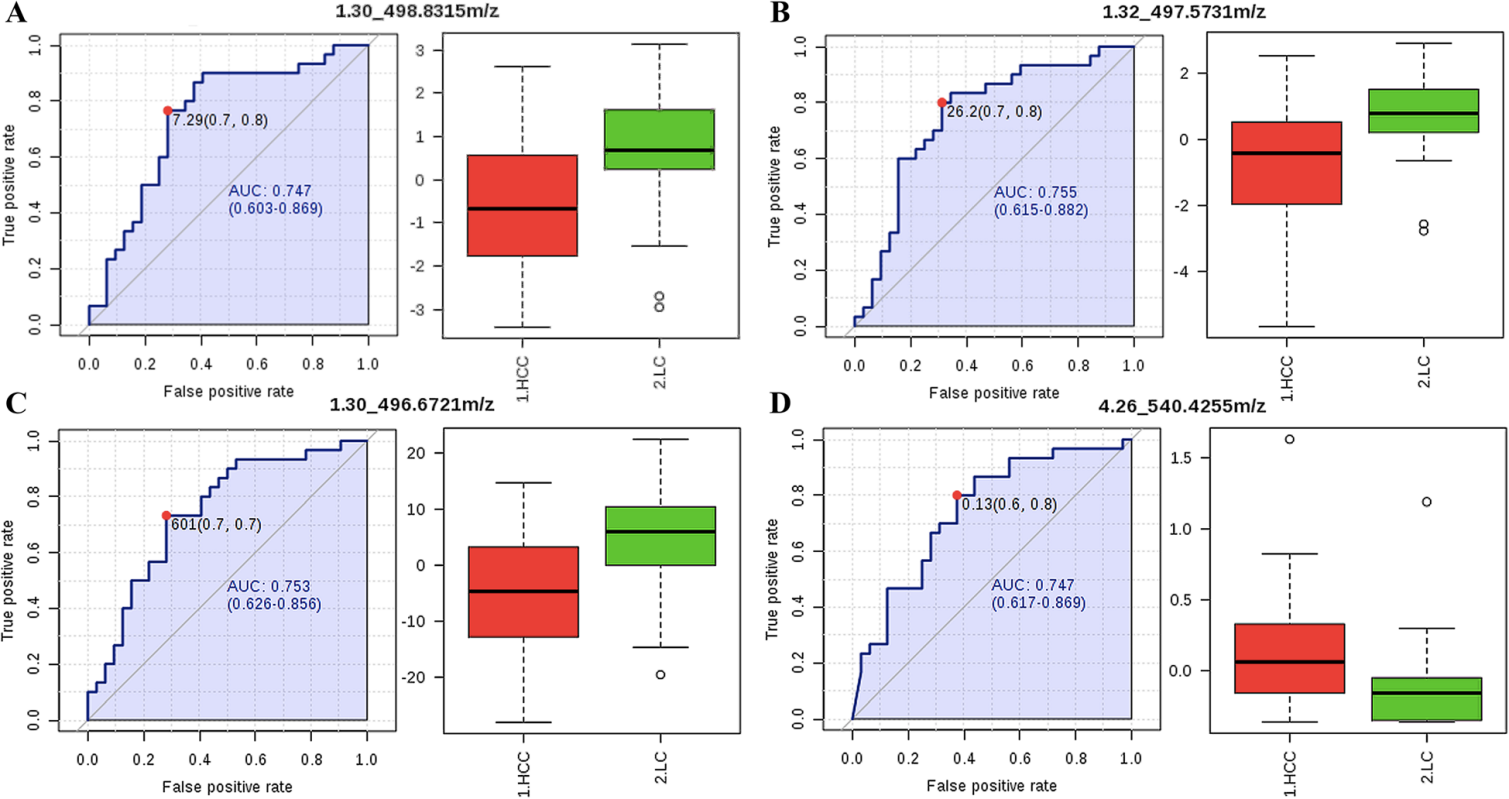
ROC curves and intensities of the differential ions in the UPLC-MS 4-peak model by RT and *m/z*. Detailed legend: ROC curves and intensities of the differential ions in the ULC-MS 4-peak model in HCC (red boxes) and LC (green boxes) patients for the ions (**a**) RT 1.30_498.8315 *m/z*; (**b**) RT 1.32_497.5731 *m/z*; (**c**) RT 1.30_496.6721 *m/z*; (**d**) RT 4.26_540.4255 *m/z*. AUC, area under the curve; HCC, hepatocellular carcinoma; LC, liver cirrhosis; CH, chronic hepatitis; HS, healthy subjects

Four lipids independently predicted HCC from LC with 65.6–84.4 % sensitivity, and 60.0–76.7 % specificity. Figure 3 shows the intensities and ROC curves of the 4 lipids in patients with HCC and LC.

**Fig. 4:**
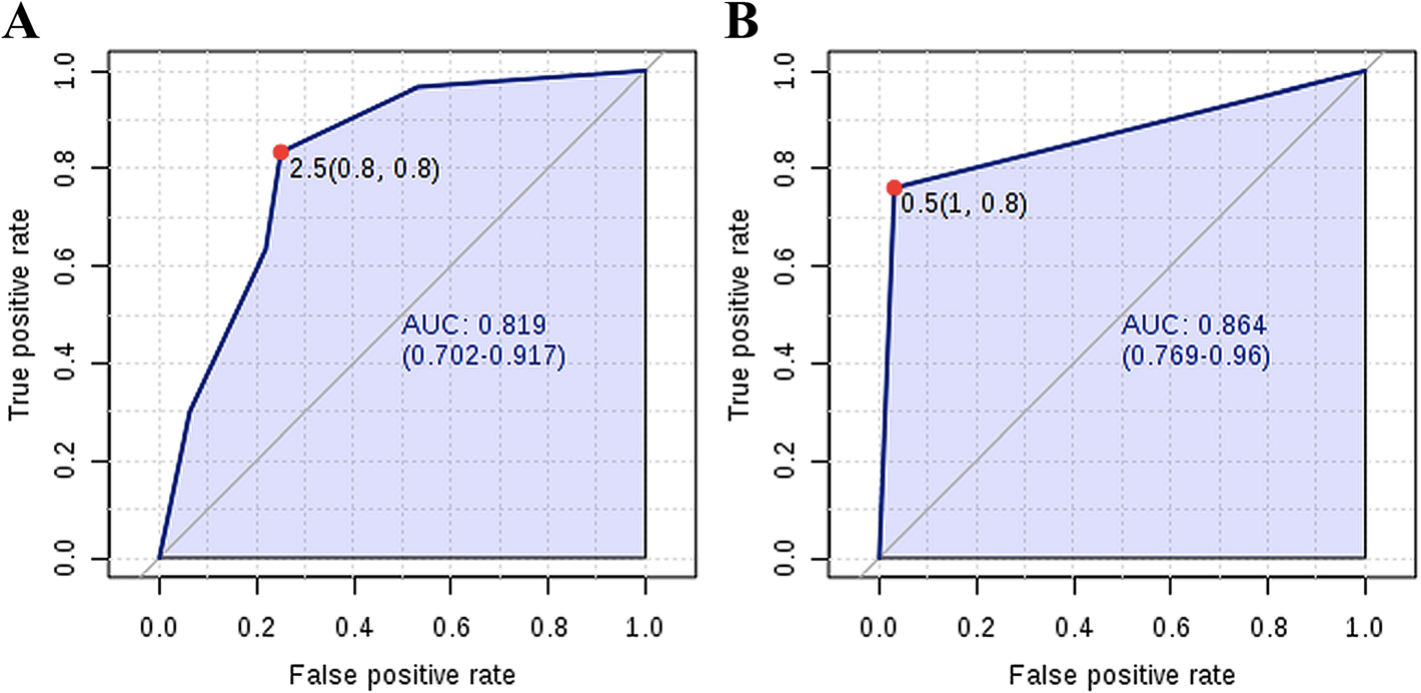
ROC curves of the UPLC-MS 4-peak algorithm in differentiating (A) HCC from LC and (B) HCC from CH. Detailed legend: AUC, area under the curve; HCC, hepatocellular carcinoma; LC, liver cirrhosis; CH, chronic hepatitis; HS, healthy subjects

Based on the efficiency of the ROC curves, cutoff values were determined for each ion. The number of “positive” ions in each sample was used to generate a 4-peak algorithm with cutoff value of at least 2 “positive” biomarkers, defined by ROC curve analysis and posterior univariate statistical validation (Fig. 4a). The 4-peak algorithm generated distinguished HCC from LC with an accuracy of 71.0 % (95 % CI 58.7–80.1 %), a sensitivity of 78.1 % (95 % CI 61.2–89.0 %), and a specificity of 63.6 % (95 % CI: 45.4–78.1 %). This algorithm successfully detected 25 of 32 HCC cases when applied to discriminate HCC from LC.

Conversely, AFP detected only 12 of 32 HCC cases from LC when cutoff value was set as 20 ng/mL, showing an accuracy of 64.5 % (95 % CI 52.1–75.3 %), a sensitivity of 37.5 % (95 % CI 22.9–54.8 %), and a specificity of 93.3 % (95 % CI: 78.7–98.2 %). In the range of 200 ng/mL, AFP detected 6 of 32 HCC cases, performing with an accuracy of 58.1 % (95 % CI 45.7–69.5 %), a sensitivity of 18.8 % (95 % CI: 8.9–35.3 %), and a specificity of 100 % (95 % CI 88.7–100.0 %) (Table 3).

**Table 3:** Sensitivity and specificity of UPLC-MS profiles, AFP and individual peaks for HCC diagnosis

Test	Sensitivity (%)	Specificity (%)	ROC AUC
HCC versus LC			
4-ion UPLC-MS model	78.1	63.6	0.819^b,c^
AFP_20 ng/mL_	37.5	93.3	0.779^d^
AFP_200 ng/mL_	18.8	100.0	0.779^d^
4-ion UPLC-MS model	75.0	83.3	0.856
+ AFP_20 ng/mL_^a^			
HCC versus CHB			
4-ion UPLC-MS model	93.8	80.0	0.864^e,f^
2-ion UPLC-MS model	96.9	80.0	0.933^g^
RT 3.40_773.5478 *n*	100.0	100.0	1.000
RT 1.87_534.3902 *m/z*	100.0	100.0	1.000
RT 6.25_369.3538 *m/z*	100.0	100.0	1.000
RT 3.45_822.5670 *m/z*	100.0	100.0	1.000
RT 3.59_770.5691 *m/z*	100.0	100.0	1.000
RT 4.23_851.6090 *m/z*	100.0	100.0	1.000
RT 3.99_826.5920 *m/z*	100.0	100.0	1.000
HCC versus HS			
4-ion UPLC-MS model	90.6	88.2	0.946

*ROC* receiver operating characteristic, *AUC* area under the curve, *HCC* hepatocellular carcinoma, *LC* liver cirrhosis, *AFP* alpha-fetoprotein, *UPLC* ultra performance liquid chromatography, *MS* mass spectrometry, *CHB* chronic hepatitis B, *RT* retention time, *n* neutral, *m/z* mass to charge ratio, *HS* healthy subjects. ^a^Combination of the 4-ion UPLC-MS model and AFP_20 ng/mL._^b^Compared with AFP_20 ng/mL_ or AFP_200 ng/mL_, *P* = 0.616. ^c^Compared with 4-ion UPLC-MS model + AFP_20 ng/mL_, *P* = 0.610. ^d^Compared with 4-ion UPLC-MS model + AFP_20 ng/mL_, *P* = 0.312. ^e^Compared with 2-ion UPLC-MS model, *P* = 0.241. ^f^Compared with individual ions, *P* = 0.047. ^g^Compared with individual ions, *P* = 0.005

The accuracy, sensitivity and specificity of HCC detection of the 4-peak algorithm was not compromised when the 6 HCC patients with Child-Pugh scores B and C were excluded from the analysis. Likewise, the HCC detection rate of the algorithm did not vary significantly when patients were stratified according to the BCLC staging system (*P* = 0.463). Very early or early HCC was detected with a sensitivity of 73.7 % (95 % CI: 51.2–88.2 %), and a specificity of 63.3 % (95 % CI: 45.5–78.1 %).

The combination of the 4-peak UPLC-MS algorithm with AFP in the range of 20 ng/mL was able to distinguish HCC from LC with an accuracy of 79.0 % (95 % CI 67.4–87.3 %), a sensitivity of 75 % (95 % CI 57.9–86.8 %), and a specificity of 83.3 % (95 % CI: 66.4–92.7 %).

### Hepatocellular carcinoma versus chronic hepatitis B

The 4 peaks independently predicted HCC from CHB with 52–90.6 % sensitivity and 68.8–86.7 % specificity. The intensities and ROC curves of the 4 lipids in patients with HCC and CHB are shown in Additional file 3. The 4-peak algorithm distinguished HCC from CHB with an accuracy of 87.1 % (95 % CI 76.6–93.3 %), a sensitivity of 93.8 % (95 % CI 79.9–98.3 %), and a specificity of 80.0 % (95 % CI 62.7–90.5 %) (Table 3).

As the ion RT 4.26_540.4255 *m/z* did not perform well in this comparison, we also tested the performance of the model using different combinations of the 4 ions. The best model was a combination of the ion RT 1.30_498.8315 *m/z* and RT 1.32_497.5731 *m/z*, which with a cutoff of at least 1 “positive” ion, detected 31 of the 32 HCC cases and distinguished HCC and CHB with an accuracy of 88.7 % (95 % CI 78.5–94.4 %), a sensitivity of 96.9 % (95 % CI 84.26–99.5 %), and a specificity of 80.0 % (95 % CI 62.7–90.5 %) (Fig. 4b).

We also looked at the whole lipidomic profile of HCC and CHB and, interestingly, 7 peaks independently predicted HCC from CHB with 100 % sensitivity and specificity (Additional file 4).

### Tentative identification of potential biomarkers

Table 4 shows the main classes and subclasses associated with the differentiating lipids found in this study.

**Table 4:** Tentative identification of potential UPLC-MS biomarkers for HCC

RT_*m/z*	Adduct	Identified result	Main class
1.30_498.8315	[M + H]+	unknown	-
1.32_497.5731	[M + H]+	unknown	-
1.30_496.6721	[M + H]+	unknown	-
4.26_540.4255	[M + H]+	unknown	-
3.40_773.5478	[M + H]+	PS(O-16:0/20:2)^a^	Glycerophosphoserines
1.87_534.3902	[M + H]+	unknown	-
6.25_369.3538	[M + H]+	unknown	-
3.45_822.5670	[M + H]+	PS(O-18:0/22:6)^b^	Glycerophosphoserines
3.59_770.5691	[M + H]+	PC(15:0/20:3)^c^	Glycerophosphocholines
4.23_851.6090	[M + H]+	PI(O-16:0/20:1)^d^	Glycerophosphoinositols
3.99_826.5920	[M + H]+	PS(O-18:0/22:4)^e^	Glycerophosphoserines

^a^(11Z,14Z); ^b^(4Z,7Z,10Z,13Z,16Z,19Z); ^c^(8Z,11Z,14Z); ^d^(11Z); ^e^(7Z,10Z,13Z,16Z)/22:5(4Z,7Z,10Z,13Z,16Z). TG, triacylglycerol; PS, phosphatidylserine; PC, phosphatidylcholine; PI, phosphatidylinositol; *m/z*, mass to charge ratio

## Discussion

Diagnosis of HCC at an early stage is essential for disease prognosis as it allows the application of curative treatments and improves patient survival.

In the present study, an UPLC-MS-based lipidomic expression signature successfully distinguished HBV-HCC cases from HBV-LC with 78.1 % sensitivity and 63.6 % specificity and provided a more precise diagnostic instrument for cirrhotic patients than conventional non-invasive biomarker detection (AFP). Our results also show that the UPLC-MS lipidomic fingerprinting discriminated serum lipidomic expression patterns among patients with HBV-HCC, HBV-LC, and CHB.

Studies on lipidomic profiling of HCC are still scarce. Moreover, key data are lacking in the few published studies, such as comprehensive description and assessment regarding patient and background liver disease characterization, group allocation and controls adequacy, and proper performance assessment of the diagnostic model, among others [14].

The results presented herein are innovative, as this study performs a robust evaluation of patients enrolled in a well-established HCC surveillance program. These patients are, therefore, well characterized as to their clinical and laboratory parameters, which ensures the adequacy of the study groups and controls, and allows an unbiased interpretation of the proposed biomarkers and their intra-group level variations.

When used as a diagnostic biomarker, AFP is expected to misdiagnose up to 40 % of HCC cases with a 20 ng/mL cutoff value [4]. In this study, however, while the UPLC-MS-based 4-peak model accurately diagnosed 25 of 32 HCC cases from LC patients, AFP performed poorly, detecting only 12 of 32 cases with a sensitivity of 37.5 % and 93.3 % specificity.

When applied to differentiate HCC in the early stages, the UPLC-MS signature detected very early or early stage HCC with 73.7 % sensitivity and 63.3 % specificity. These data show the potential applicability of UPLC-MS for screening biomarkers for early diagnosis of HCC.

Patients at high risk of HCC development should be screened semi annually using ultrasonography (US). It is known, however, that in most cases US has only acceptable diagnosis accuracy with sensitivity ranging from 58 to 89 % and specificity greater than 90 % [4, 15, 16]. Furthermore, US effectiveness for detecting early-stage HCC is even lower, with a sensitivity of only 63 % [17]. The accuracy of the proposed UPLC-MS 4-peak model for HCC screening and the actual gain in the detection rate need to be further evaluated on larger studies. Nonetheless, the use of this model might improve HCC surveillance and diagnosis, especially in resource-limited regions where patients may have difficult access to US and higher resolution imaging techniques such as CT scan and magnetic resonance. A lipidomic biomarker and/or profile could be, in turn, detected through a simple, inexpensive and widely accessible enzyme immunoassay or chemiluminescence assay, which would represent a significant reduction on HCC screening costs.

HBV infection can lead to HCC in the absence of cirrhosis. Although little is known about the clinical and epidemiological aspects of HCC in Brazil [18], data from other regions show that 20 to 30 % of patients with HBV-related HCC do not present with LC [19]. In this study the rate of HCC in the absence of cirrhosis was 12.5 %. The UPLC-MS 4-peak detected HCC from CHB patients with of 93.8 % sensitivity and a specificity of 80.0 %. Furthermore, it was observed that some peaks not included in the first model could differentiate HCC and CHB with 100 % sensitivity and specificity.

We performed a tentative and preliminary identification of the differentially expressed peaks. At this point we have identified 3 glycerophosphoserines, 1 glycerophosphocholine and 1 glycerophosphoinositol, all in significantly lower levels in HCC patients.

Previous studies also have shown lower levels of glycerophosphocolines in HCC patients, which are the most abundant phospholipid in mammalian cellular membranes [11]. This under expression may result from the inflammatory response and consequent higher consumption of these lipids [20, 21]. CHB infection has been associated with alterations in lipid metabolism and a recent study showed that HBV infection altered the metabolic gene expression in a human liver-chimeric mouse model by altering bile acid and cholesterol metabolism as a consequence of impaired bile acid uptake [22].

## Conclusions

Our findings suggest that UPLC-MS lipidomic fingerprinting may be a powerful tool for the identification of diagnostic biomarkers and models for hepatitis B virus-related HCC. These data showed that the lipid fingerprinting in HCC patients selected a number of lipids that should be functionally investigated to elucidate the pathogenesis of the disease. This technique and the selected peaks show a great potential to improve HCC surveillance in patients with LC and CHB.

## Additional files

Additional file 1:
**PCA scores plot based on the UPLC-MS profiling data for the studied groups.** The score plots show the first, second and third principal components. Each dot in the plot represents a patient according to its group. HCC, hepatocellular carcinoma; LC, liver cirrhosis; CH, chronic hepatitis; HS, healthy subjects. (TIF 1120 kb) Additional file 2:
**Distribution of HCC patients according to BCLC staging system.** (TIF 62 kb) Additional file 3:
**ROC curves and intensities of the differential ions in the UPLC-MS 4-peak model by RT and** ***m/z.*** ROC curves and intensities of the differential ions in HCC (red boxes) and CH (green boxes) for (A) RT 1.30_498.8315 *m/z*; (B) RT 1.32_497.5731 *m/z*; (C) RT 1.30_496.6721 *m/z*; (D) RT 4.26_540.4255 *m/z*. AUC, area under the curve; HCC, hepatocellular carcinoma; LC, liver cirrhosis; CH, chronic hepatitis; HS, healthy subjects. (TIF 4244 kb) Additional file 4:
**ROC curves and intensities of the differential ions by RT and** ***m/z.*** ROC curves and intensities of the differential ions in HCC (red boxes) and CH (green boxes) for (A) RT 3.40_773.5478n; (B) RT 4.23_851.6090 *m/z*; (C) RT 3.59_770.5691 *m/z*; (D) RT 3.45_822.5670 *m/z*; (E) RT 6.25_369.3538 *m/z*; (F) RT 1.87_534.3902 *m/z*; (G) RT 3.99_826.5920 *m/z*. AUC, area under the curve; HCC, hepatocellular carcinoma; LC, liver cirrhosis; CH, chronic hepatitis; HS, healthy subjects. (TIF 7531 kb)
