# Supplementary figures and images for: Serum lipidomic profiling as a useful tool for screening potential biomarkers of hepatitis B-related hepatocellular carcinoma by ultraperformance liquid chromatography–mass spectrometry
Source: BMC Cancer. 2015 Dec 18;15:985. doi: 10.1186/s12885-015-1995-1 (PMC4683945; doi:10.1186/s12885-015-1995-1)

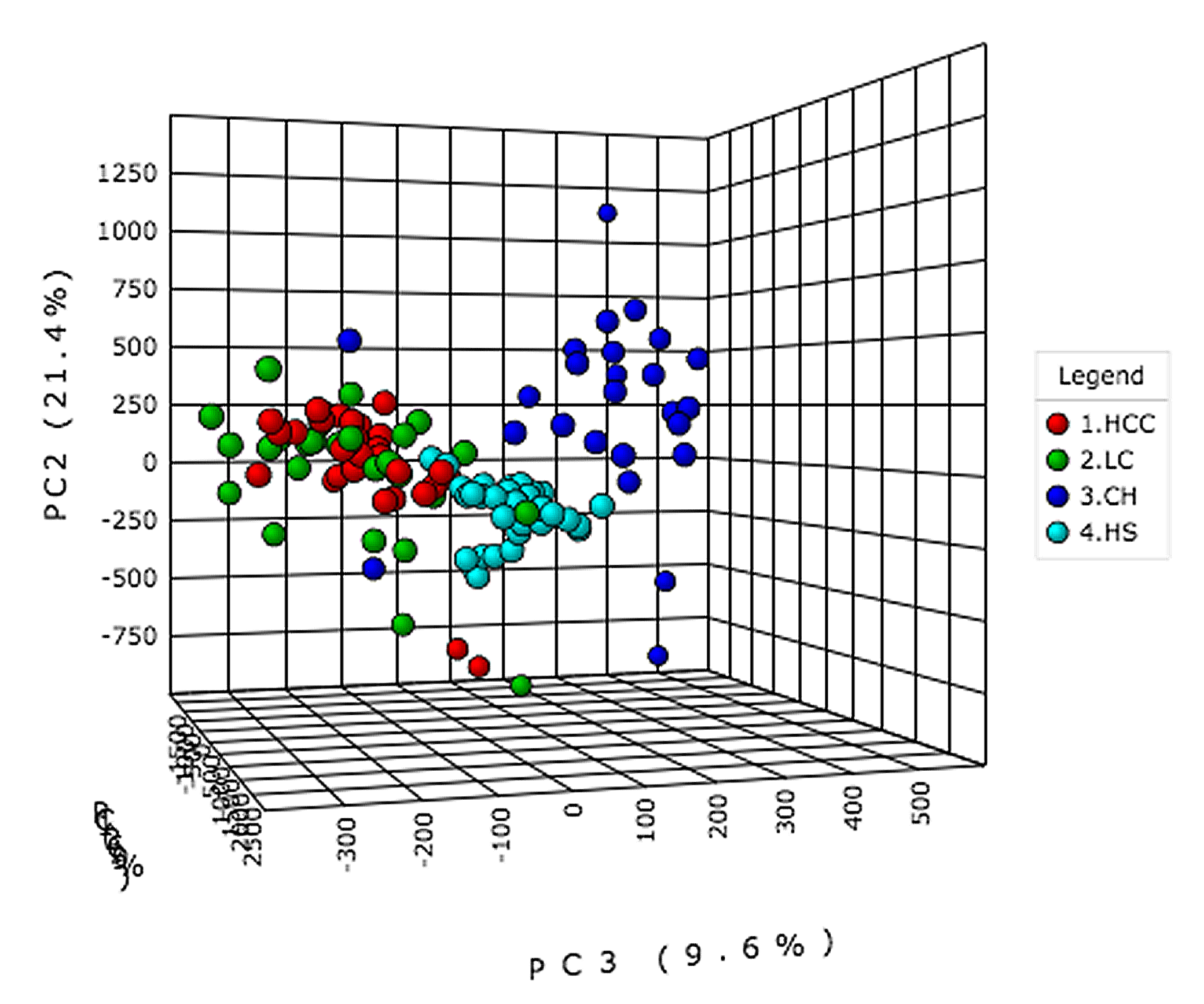

Supplement: Additional file 1: — PCA scores plot based on the UPLC-MS profiling data for the studied groups. The score plots show the first, second and third principal components. Each dot in the plot represents a patient according to its group. HCC, hepatocellular carcinoma; LC, liver cirrhosis; CH, chronic hepatitis; HS, healthy subjects. (TIF 1120 kb) [file 12885_2015_1995_MOESM1_ESM.tif]

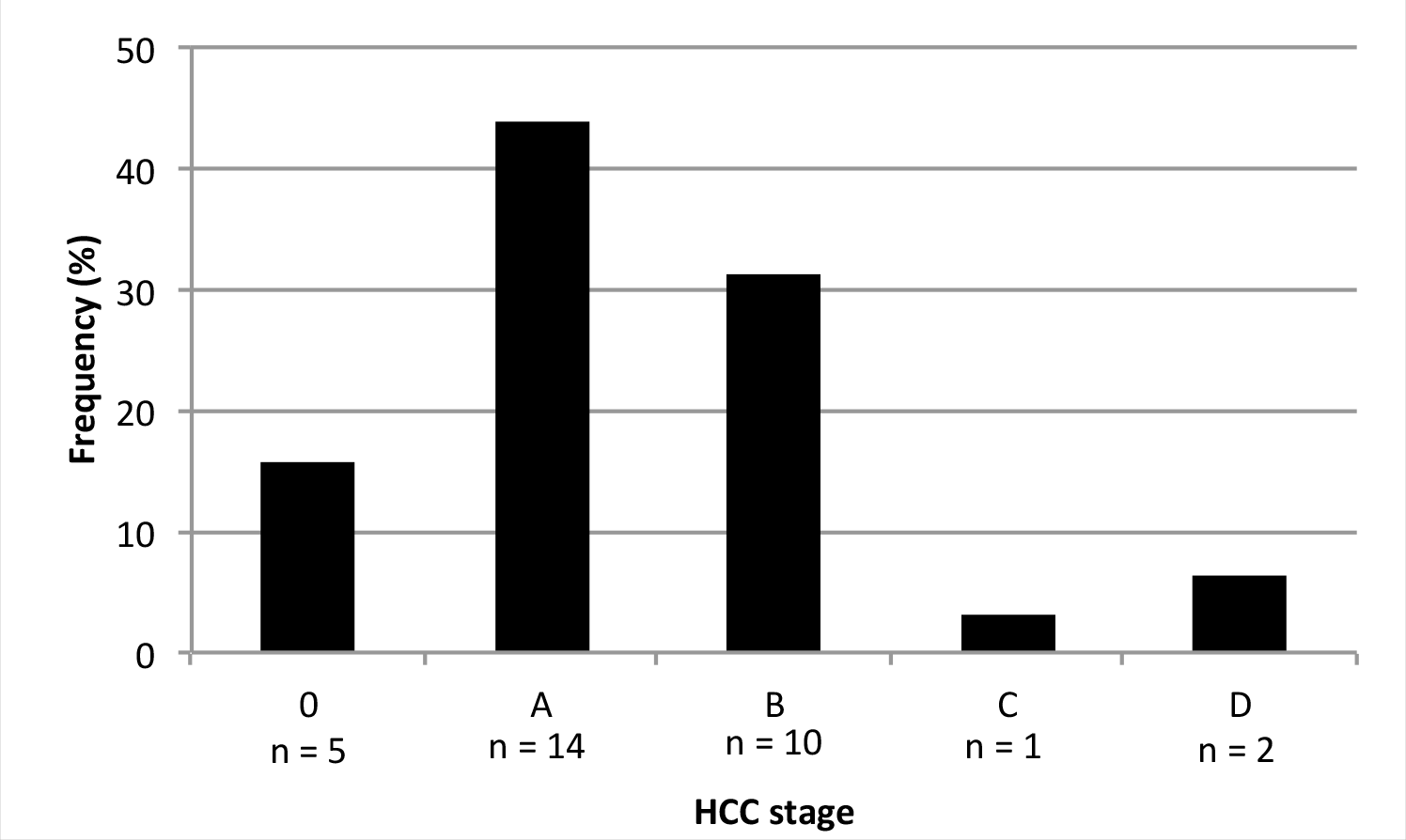

Supplement: Additional file 2: — Distribution of HCC patients according to BCLC staging system. (TIF 62 kb) [file 12885_2015_1995_MOESM2_ESM.tif]

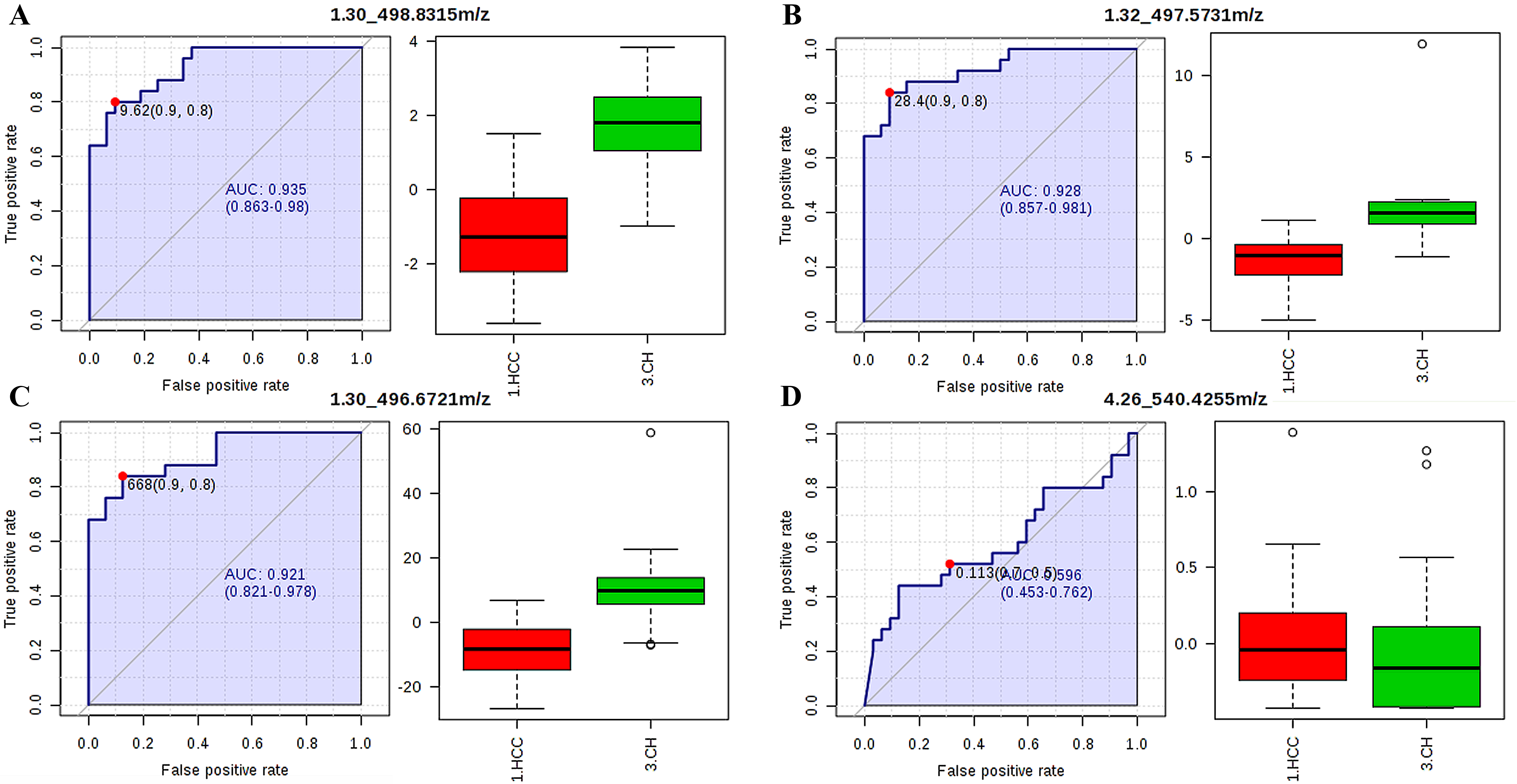

Supplement: Additional file 3: — ROC curves and intensities of the differential ions in the UPLC-MS 4-peak model by RT and m/z. ROC curves and intensities of the differential ions in HCC (red boxes) and CH (green boxes) for (A) RT 1.30_498.8315 m/z; (B) RT 1.32_497.5731 m/z; (C) RT 1.30_496.6721 m/z; (D) RT 4.26_540.4255 m/z. AUC, area under the curve; HCC, hepatocellular carcinoma; LC, liver cirrhosis; CH, chronic hepatitis; HS, healthy subjects. (TIF 4244 kb) [file 12885_2015_1995_MOESM3_ESM.tif]

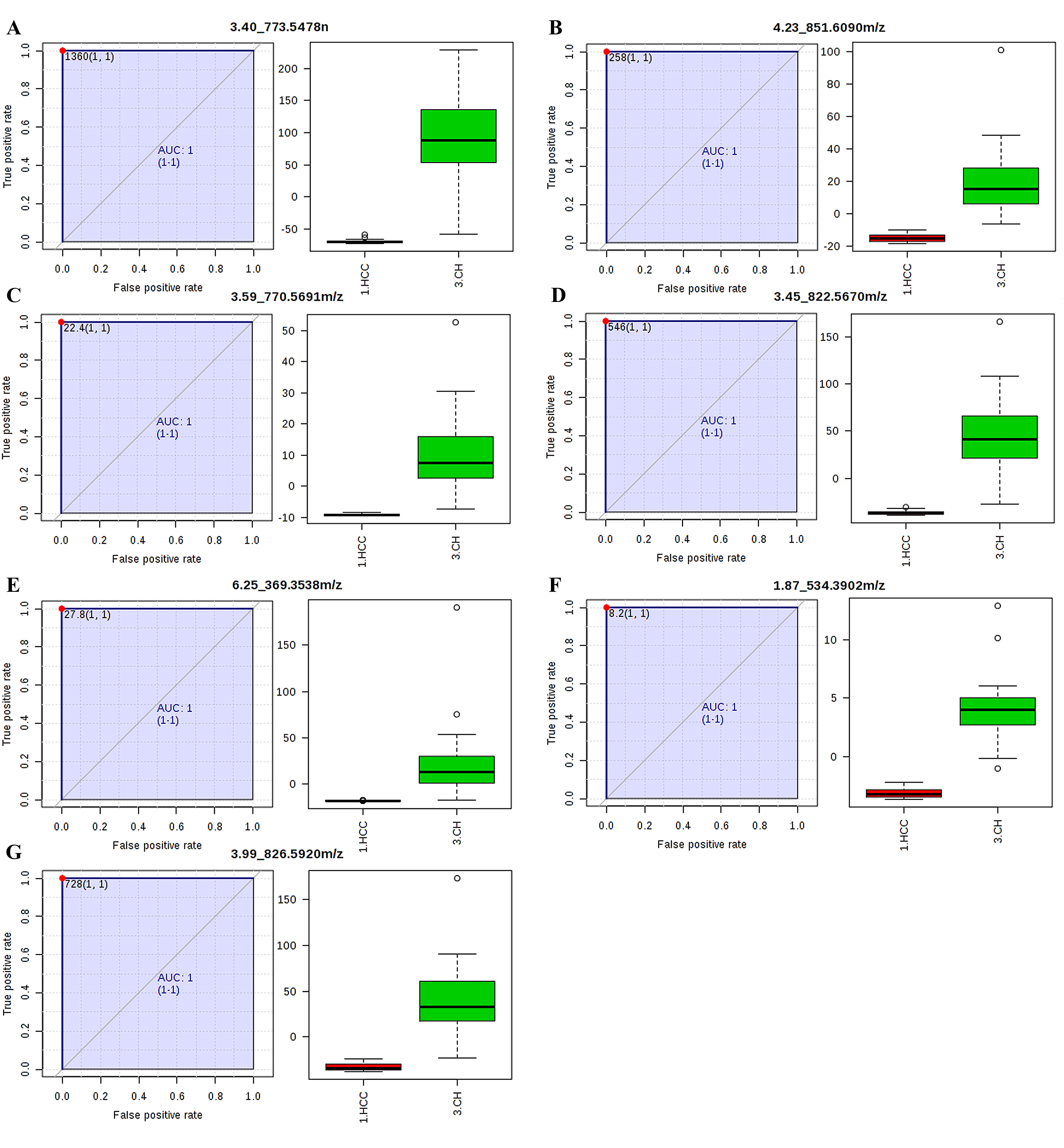

Supplement: Additional file 4: — ROC curves and intensities of the differential ions by RT and m/z. ROC curves and intensities of the differential ions in HCC (red boxes) and CH (green boxes) for (A) RT 3.40_773.5478n; (B) RT 4.23_851.6090 m/z; (C) RT 3.59_770.5691 m/z; (D) RT 3.45_822.5670 m/z; (E) RT 6.25_369.3538 m/z; (F) RT 1.87_534.3902 m/z; (G) RT 3.99_826.5920 m/z. AUC, area under the curve; HCC, hepatocellular carcinoma; LC, liver cirrhosis; CH, chronic hepatitis; HS, healthy subjects. (TIF 7531 kb) [file 12885_2015_1995_MOESM4_ESM.tif]
